# Decoding the landscape of bispecific antibodies in breast cancer: insights from a comprehensive trial analysis

**DOI:** 10.3389/fimmu.2026.1851581

**Published:** 2026-06-09

**Authors:** Siyuan Yang, Zhirui Chuan, Shoutao Yang, Jianyun Nie

**Affiliations:** 1 Department of Breast Surgery, Peking University Cancer Hospital Yunnan Hospital, Yunnan Cancer Hospital, Third Affiliated Hospital, Kunming Medical University, Kunming, Yunnan, China; 2 Department of Ultrasound, Peking University Cancer Hospital Yunnan Hospital, Yunnan Cancer Hospital, Third Affiliated Hospital of Kunming Medical University, Kunming, Yunnan, China; 3 Department of Labor Union, Peking University Cancer Hospital Yunnan Hospital, Yunnan Cancer Hospital, Third Affiliated Hospital, Kunming Medical University, Kunming, Yunnan, China

**Keywords:** bispecific antibody, breast cancer, clinical trials, immunotherapy, PD-1/PD-L1

## Abstract

Bispecific antibodies (bsAbs) represent a novel therapeutic strategy for breast cancer by leveraging diverse mechanisms of action (MOA) to overcome the limitations of conventional therapies. However, systematic analyses of bsAbs in breast cancer remain scarce. Based on the Trialtrove database (search up to September 7, 2025), we identified 193 eligible clinical trials investigating bsAbs in breast cancer. Our analysis reveals a significant surge in trials beginning in 2019, peaking in 2023. While most trials are in early phases (73.58%), the emergence of Phase III studies since 2023 signals a maturation trend. Immune checkpoint modulation dominates (64.77%), with PD-1/PD-L1 blockade as the leading strategy, followed by HER2-targeting bsAbs. Completed trials with positive outcomes highlight PD-1-based and HER2-targeted bsAbs as the most promising candidates. These findings provide a data-driven roadmap for bsAbs development in breast cancer, emphasizing the need to optimize target combinations and identify predictive biomarkers.

## 1 Introduction

Bispecific antibodies (bsAbs) offer a novel therapeutic strategy for breast cancer by leveraging diverse mechanisms of action to overcome the limitations of conventional therapies (1, 2). These strategies encompass immune checkpoint modulation, immune cell engagers, and signaling pathway blockade (3). Despite the growing interest in bsAbs, systematic analyses of their clinical development landscape in breast cancer remain limited. In addition, the development of bsAbs faces multiple challenges, including target combination selection, tumor heterogeneity, on-target off-tumor toxicity, and the lack of validated predictive biomarkers to guide patient stratification. Addressing these challenges is critical for advancing bsAbs toward clinical maturity in breast cancer. Here, we provide a comprehensive analysis of bsAbs clinical trials in breast cancer using the Trialtrove database, aiming to delineate trends, identify key targets, and inform future drug development strategies.

## 2 Materials and methods

**Figure 1:**
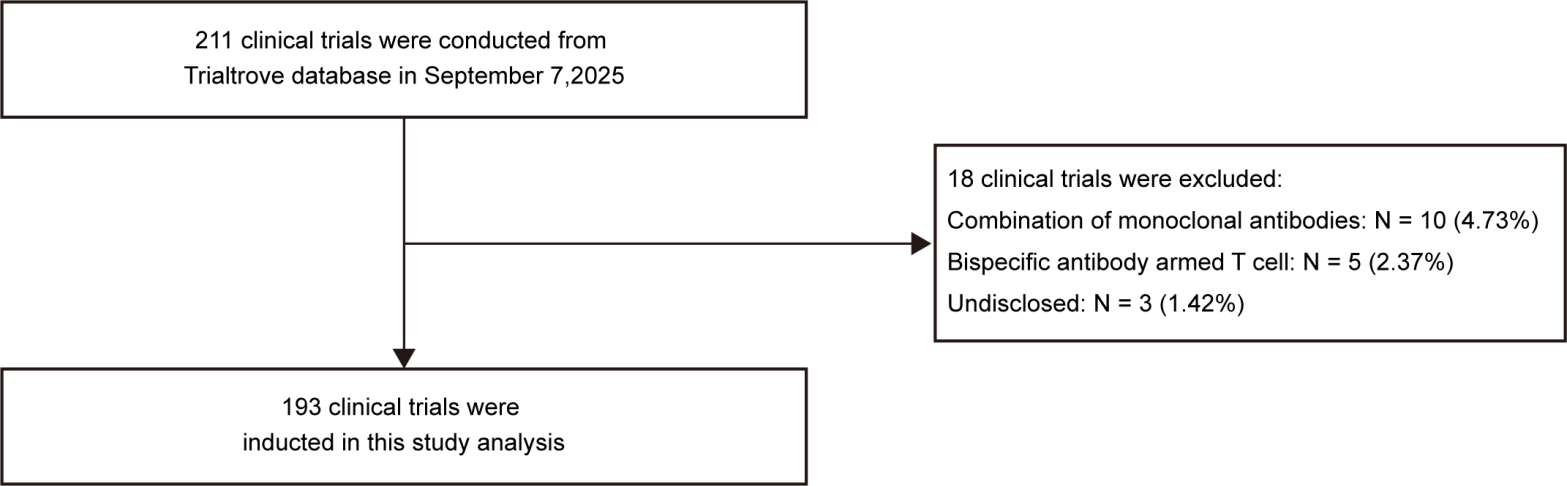
Flow chart of study selection.

In this study, the Trialtrove database was used for a comprehensive search. The search was conducted from database inception up to September 7, 2025. The compound search terms included: [(Disease is Oncology: Breast cancer)] AND [(Drug Type is Biological > Protein > Antibody > Bispecific antibody) OR (Drug Type is Biological > Protein > Antibody > Cell engager, bispecific)]. To ensure the accuracy of the research team’s conclusions, the source data were independently examined and double-checked by two investigators. After the exclusion of studies without detailed information and not included bsAbs, 193 eligible trials were identified eventually (**Figure 1**).

## 3 Result

**Figure 2:**
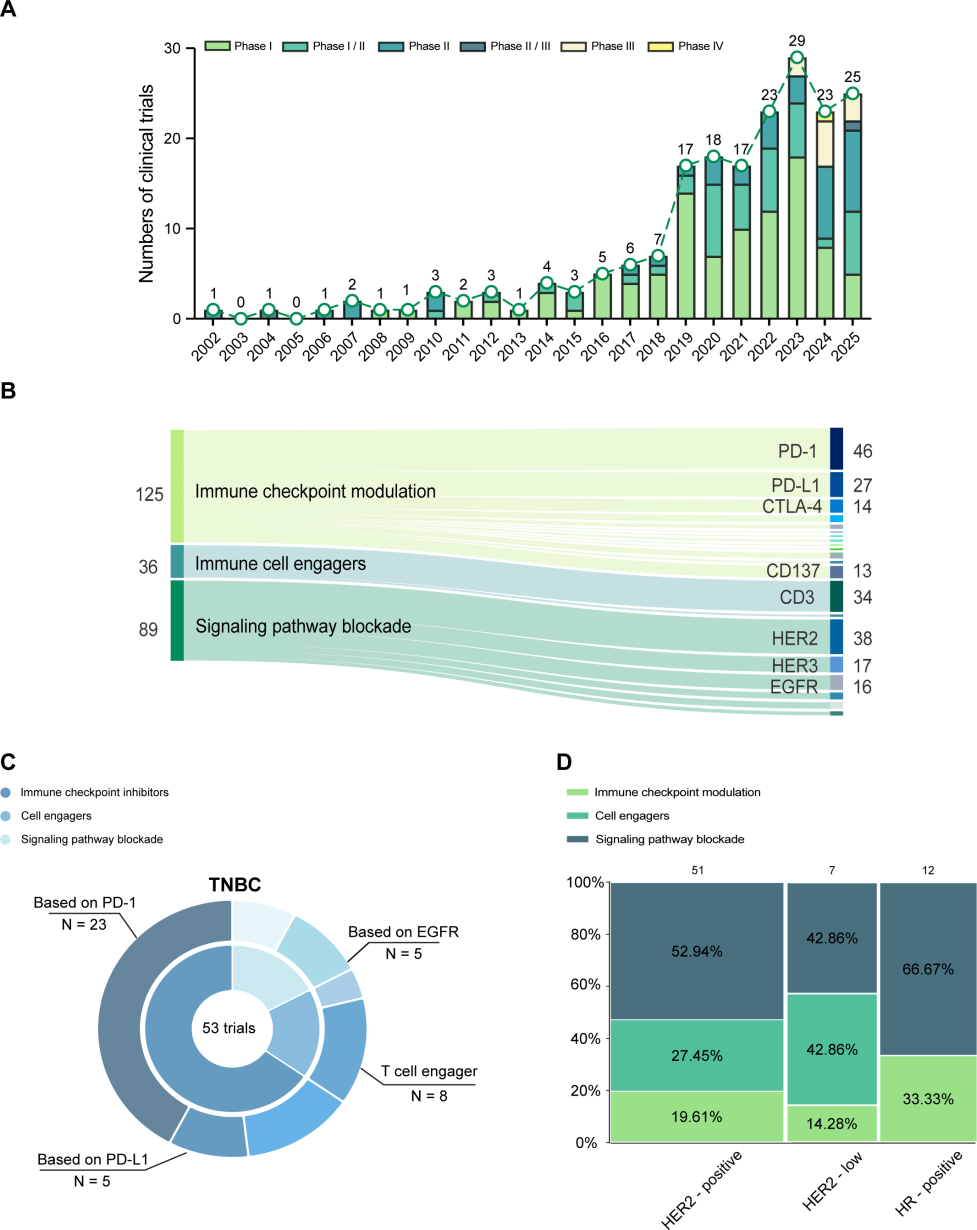
Landscape of clinical trials of bsAbs in breast cancer. **(A)** Temporal Trends in clinical trials of bsAbs in breast cancer. **(B)** Targets distribution of bsAbs clinical trials in breast cancer. **(C)** Trial distribution of bsAbs clinical trials in different subtypes of breast cancer. **(D)** Trial distribution of bsAbs clinical trials of TNBC.

Our analysis revealed a significant surge in clinical trials investigating bsAbs therapy for breast cancer starting in 2019, peaking in 2023 (**Figure 2A**). While the majority of trials remain in early phases (Phase I- I/II: 73.58%), reflecting an exploratory landscape, the emergence of Phase III trials since 2023 (n = 10) signals a maturation trend toward efficacy and safety validation. This translational momentum toward clinical maturity in breast cancer is consistent with the overall development trend in the solid tumor bsAbs field (4), highlighting the increasing value of bsAbs in breast cancer.

Regarding MOA, target selection is a critical determinant in trial design. We first observed that bsAbs based on immune checkpoint modulation account for the majority of trials (125 trials, 64.8%), with PD-1/PD-L1 blockade representing 23.83% (46 trials) and 13.98% (27 trials) respectively, followed by CTLA-4 blockade (14 trials). Meanwhile, HER2-targeting bsAbs account for 38 trials and demonstrate substantial potential in signal transduction pathway blockade (**Figure 1B**), with the majority (n = 32) being dual-epitope constructs targeting distinct domains of the HER2 (38 trials), demonstrate substantial potential in signal transduction pathway blockade (**Figure 2B**). When further analyzed by breast cancer subtype, PD-1/PD-L1-targeted bsAbs show promise in triple-negative breast cancer (TNBC) (23/5 trials) (**Figure 2C**), where validated targets are scarce. In contrast, HER2-positive breast cancer provides an ideal model for bsAbs designed for multi-domain signal inhibition (51 trials) (**Figure 2D**). Notably, the integration of bsAbs with antibody-drug conjugates (ADCs)—such as HER2-targeted bsADCs (8 trials) —may amplify therapeutic efficacy by enabling precise delivery of cytotoxic payloads while circumventing resistance mechanisms inherent in monotherapy, particularly in HER2-low subtype. These findings underscore that the next generation of bsAbs should focus on target selection informed by the molecular heterogeneity of subtypes, thereby enabling better patient stratification and increasing the probability of success in clinical trials.

**Figure 3:**
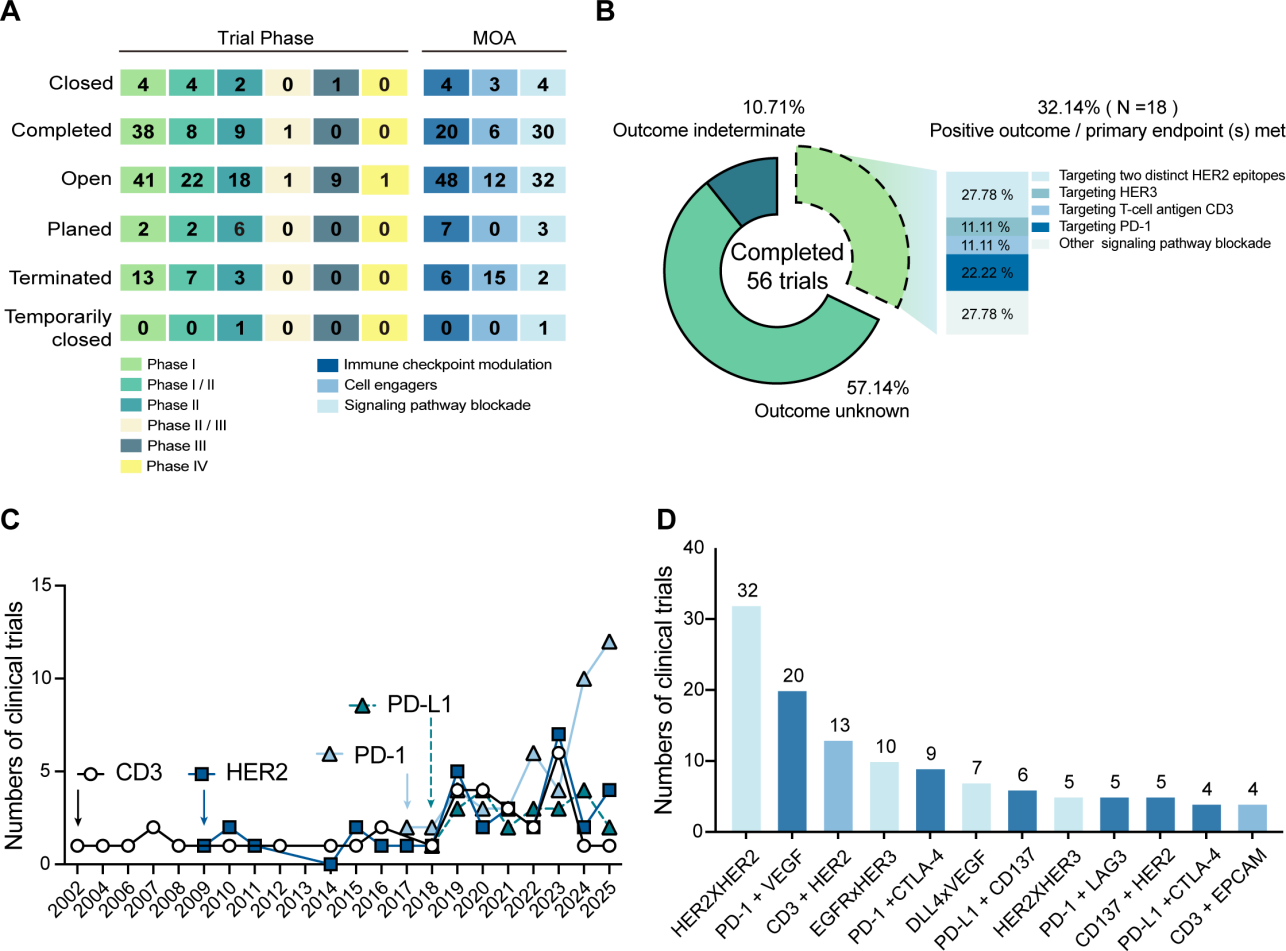
Landscape of clinical trials of bsAbs in breast cancer. **(A)** Distribution of bsAbs clinical trials by Trial status and MOA in breast cancer. **(B)** The outcome of completed trials. **(C)** Trends in bsAbs targers by year. **(D)** Target combinations of bsAbs in breast cancer.

At present, most of clinical trials are completed (56 trials, 29.0%) or open (92 trials, 47.7%). Correspondingly, bsAbs based on immune checkpoint modulation account for a considerable proportion in both statuses (35.7% in completed, 52.2% in open). Surprisingly, in the terminated clinical trials, only a very small proportion (1/23 trials,4.3%) was due to the lack of efficacy, and more were due to the drug strategy shift/pipeline reprioritization (16/23 trials). This distribution feature also indirectly confirms that this therapy still has potential value worth paying attention to in the R&D pipeline. Its termination more reflects the dynamic optimization of industry strategies rather than the lack of prospects for the therapy itself. By analyzing the positive results (18 trials in positive outcome/primary endpoint (s) met), we found that PD-1-based bsAbs demonstrated equal importance to bispecific antibodies that blocked HER2 domain II and domain IV (**Figure 3B**). Moreover, bsAbs targeting PD-1 demonstrated favorable efficacy in both early and advanced breast cancer, while maintaining good treatment tolerance (**Supplementary Table 1**). This suggests that PD-1 and HER2 currently represent optimal options, whereas the combined target necessitates additional investigation, primarily due to spatial and temporal heterogeneity with breast cancer.

The development of bsAbs has undergone a significant evolution, shifting from early strategies centered on CD3-based T-cell engagers—which, despite two decades of research from 2002, have been limited by off-target toxicity and modest efficacy (5)—to immune checkpoint modulation (**Figure 3C**). Therefore, the development of bispecific CD3 T cell engagers should take into account the selection of T cells, the recruitment of irrelevant T cell subsets such as exhausted T cells or the recruitment of immunosuppressive T cell populations such as regulatory T (Treg) cells should be avoided, as these may undermine therapeutic efficacy and fail to mount an effective immune response. In contrast, clinical trials of HER2-targeting and PD-1/PD-L1-based bsAbs have steadily increased, with those centered on PD-1 representing the largest proportion (**Figure 3C**). This growth can be attributed to two main factors. First, PD-1/PD-L1 monoclonal antibodies have been validated as an effective immunotherapy backbone, and their bispecific formats enable synergistic checkpoint inhibition while maintaining a manageable safety profile. Second, the molecular heterogeneity of HER2 signaling has driven the development of diverse bsAbs that achieve superior signaling blockade compared with conventional monoclonal antibodies. Consequently, the current bsAb development landscape is characterized by greater diversity in target selection.

Among these, PD-1 has emerged as the unequivocal leader since its rise around 2018. At present, the research and development of bsAbs is more focused on the optimization of target combination strategies. In terms of the scale of target combinations, the number of combined studies of PD-1 and VEGF is slightly less than that of HER2 bsAbs, while the combination of PD-1 with other targets (e.g. CTLA-4 and LAG3) also shows a growth trend (**Figure 3D**). In addition, more targets have been integrated into immune cell engager bsAbs constructs, including Trop2, CEACAM5, placental cadherin, EpCAM, and EGFR. BsAbs that block BsAbs that block the immune checkpoint receptor T cell immunoreceptor with immunoglobulin and ITIM domains (TIGIT) or its ligand are also being evaluated recently (6). Given their rapid recent growth, diverse combination partners, and emerging biomarker support, PD-1-centered bsAbs may be more promising for development, though their optimal partner remains uncertain. Searching for precise biomarkers that can predict which patients will respond to a given treatment is an important part of precision medicine and helps provide the most suitable “partner” for breast cancer.

## 4 Discussion

In this study, we systematically analyzed 193 clinical trials of bsAbs in breast cancer using the Trialtrove database. Our findings first reveal that the bsAbs landscape in breast cancer has shifted from early CD3-based T-cell engagers toward immune checkpoint modulation, with PD-1/PD-L1 blockade emerging as the dominant strategy (64.8% of trials). This trend aligns with the broader solid tumor bsAbs field, where PD-1/PD-L1 and CTLA-4 bispecifics are at the forefront of ongoing development (7, 8). However, only 38% of bsAbs trials in solid tumors (n = 258 out of 681) specified a biomarker inclusion criterion (4). The absence of predictive biomarkers therefore limits the ability to identify patients most likely to benefit from specific bsAbs regimens. Even so, our analysis points to another direction—bispecific antibody-drug conjugates (bsADCs). The HER2×TROP2 bsADC (e.g., OBI−201) exemplifies this paradigm, as it overcomes low antigen expression and bypasses resistance mechanisms inherent to monospecific ADCs, offering a particularly attractive strategy for HER2−low breast cancer. While PD−1/PD−L1−based (46 trials) and HER2−targeting bsAbs (38 trials) continue to advance, bsADCs represent a next−generation modality with the greatest translational potential.

Despite the promise of bsADCs, the challenge of predictive biomarkers remains critical and is now being addressed by emerging evidence in breast cancer. In HER2-positive breast cancer, the HER2-targeted bispecific antibody KN026 has been evaluated with biomarker exploration: a phase I study identified that co-amplification of HER2 (vs. no co-amplification) of CDK12 was associated with better response to KN026 (binds to two distinct HER2 epitopes) was a promising biomarker in predicting better response to KN026 (9). Spatial molecular analyses further revealed that high expression of CALML5, TFAP2B, and ERBB2 in tumor cells at baseline correlated with objective response to KN026 (10). In TNBC, patients with TASOR2 or MST1R mutation had a significantly lower rate of pathological complete response (pCR) after receiving cadonilimab (a PD-1/CTLA-4 bispecific antibody) (11). Therefore, identifying more robust and representative biomarkers will be essential to optimize bsAbs efficacy and enable precise patient stratification.

Building upon the need for predictive biomarkers, optimizing target combinations represents another critical direction for bsAbs development in breast cancer. Given the molecular heterogeneity of HER2 signaling and the substantial patient population with HER2-positive breast cancer, HER2-targeting bsAbs have attracted extensive research interest. Beyond the dual-epitope HER2×HER2 bsAbs (e.g., anbenitamab/KN026) that promote receptor clustering and internalization (12, 13), emerging formats include HER2×CD3 T-cell engagers that redirect cytotoxic T cells to HER2-expressing tumor cells (14), HER2×HER3 bispecific antibodies that block both homodimer and heterodimer signaling (15), and HER2×CD137 costimulatory bsAbs that enhance T-cell activation within the tumor microenvironment (16). All these diverse MOAs have demonstrated encouraging preclinical and early clinical activity. Therefore, selecting the optimal combination partner for HER2 in the context of breast cancer is of paramount importance. Moreover, the expression of HER2 in non-tumor tissues such as cardiomyocytes represents a major cause of on-target off-tumor toxicity (17), a consideration that must inform the choice of bispecific partners to minimize adverse effects while maximizing therapeutic index.

Beyond HER2-targeting strategies, PD-1/PD-L1-based bsAbs have demonstrated encouraging efficacy in TNBC (18–20). These findings further underscore that, across distinct molecular subtypes of breast cancer, the tumor microenvironment (TME) and immune status directly determine the therapeutic efficacy of immune checkpoint-centered bsAbs. As summarized in the KN046 study, TNBC with higher PD-L1 expression, has been demonstrated to benefit from immune checkpoint inhibitor treatment (19). Consequently, integrating immune activation status into clinical trial design—rather than relying solely on histological classification—will be essential to optimize patient selection and maximize therapeutic outcomes for TNBC patients receiving bsAb-based immunotherapy. However, tumor heterogeneity is a key variable beyond conventional biomarker measurements. Within a single tumor, the distribution of PD-L1 expression and TILs varies substantially across regions (21, 22). Moreover, the redundancy of immune checkpoint pathways—where tumors upregulate alternative inhibitors (e.g., CTLA-4, LAG3, TIM-3) upon PD-1 blockade—represents adaptive heterogeneity that compromises single-mechanism bsAbs (23). Bispecific antibodies targeting PD-1/PD-L1 together with VISTA, CTLA-4, or LAG3 may overcome this adaptive resistance. Indeed, an Fc-based bsAb targeting VISTA and PD-L1 induced higher levels of breast cancer cell lysis and pro-inflammatory cytokines (IFN-γ, TNFα, Granzyme B) than monotherapy or combination therapy with parental antibodies (24). Rather than viewing tumor heterogeneity merely as a constraint, we see it as an opportunity to drive rational target combinations. This requires integrating spatial immune profiling, dynamic biomarker monitoring, and mechanism-driven trial designs into early-phase development—moving beyond the mere stacking of targets.

At present, the integration of bsAbs with antibody-drug conjugates (bsADCs) is an emerging trend. Evidence indicates that bsADCs enhance antitumor activity through dual-targeted drug delivery (25). Notably, a novel HER2 × TROP2 bispecific ADC (OBI-201) has demonstrated potent antitumor activity across multiple tumor models, irrespective of HER2 and TROP2 expression levels (26). These findings suggest that optimizing bsAb architecture—whether through target selection, immune engagement, or conjugation with cytotoxic payloads—can further enhance therapeutic efficacy and potentially overcome the limitations imposed by low target antigen expression. Therefore, continued investigation into bsADC platforms is warranted to expand treatment options for patients with historically underserved breast cancer subtypes. Despite manual verification of all original publications, early-phase trials and conference abstracts often incompletely report biomarker or patient subgroup data. Moreover, the lack of patient-level data prevents independent assessment of biomarker–response correlations.

## 5 Conclusion

In conclusion, bsAbs have introduced a new treatment paradigm for advanced breast cancer, with emerging data suggesting potential utility in early-stage settings. While the recent initiation of Phase III trials marks progress, the path to clinical maturity remains challenging. Tumor heterogeneity and antigen escape may complicate target selection and contribute to resistance (27), underscoring the need for predictive biomarkers. Notably, although PD-1 has emerged as a leading target in bsAbs development, identifying its optimal combination partner remains an open question. Future research should prioritize biomarker-driven strategies to refine target combinations and enable precise patient stratification.

## Data Availability

The original contributions presented in the study are included in the article/**Supplementary Material**. Further inquiries can be directed to the corresponding authors.
