# Supplementary material for: Decoding the landscape of bispecific antibodies in breast cancer: insights from a comprehensive trial analysis
Source: Front Immunol. 2026 Jun 9;17:1851581. doi: 10.3389/fimmu.2026.1851581 (PMC13287128; doi:10.3389/fimmu.2026.1851581)
Supplement: Supplementary file 1 [file DataSheet1.pdf]

Supplementary table 1: Key Clinical Trial Results of Representative Bispecific Antibodies.

BsAbs	NCT number	Targets	Efficacy	TRAEs
Anbenitamab	NCT04881929 []	HER2×HER2	tpCR 56.7%, bpCR 60%, ORR 90.0%	TEAEs 100%, serious TEAEs 53.3%
	NCT04165993	HER2×HER2	ORR 76.4%, CBR 85.5%, mPFS 27.7 months	Serious TEAEs63.2%
	NCT05838066	HER2×HER2	ORR 28.1%, mPFS 6.8 mons	Pyrexia (23.8%), diarrhea (22.2%), aspartate aminotransferase increased (22.2%), serious TEAEs 6.34%.
	NCT03619681	HER2×HER2	DCR 69.8%, mPFS 5.6 mons, CR 1.6%, PR 23.8%, SD 44.4%	/
Cadonilimab	NCT03261011	PD-1 × CTLA-4	ORR 13.4%, DoR 12.9 months	Serious TRAEs 13.4%. Any grade irAEs 44.5%, with grade ≥3 irAEs 6.7%
	NCT05915481	PD-1 × CTLA-4	ORR 23.8%, PFS 7.2 months, OS 10.0 months, Local control rates (6 month) 98.4%, Local control rates (12 month) 93.0%	TRAEs 38.1%, with grade 3 TRAEs 3.2%

BsAbs	NCT number	Targets	Efficacy	TRAEs
Cadonilimab	ChiCTR2200067005	PD-1 × CTLA-4	tpCR 65.5%, bpCR 72.4%, ORR 93.1%, DCR 100%	TRAEs 100%, with 51.7% serious TRAEs 6.9%. irAEs 48.0%, serious irAEs 3.4%.
Zanidatamab	NCT04224272	HER2(D2) × HER2(D4)	PFS (6 mon) 67%, mPFS 11.7mons	Only one serious TEAE.
Zenocutuzumab	NCT03321981	HER2×HER3	CBR 35.1%， ORR 18.9%,CR 2.7%， PR 16.2% ， SD 59.5%	Diarrhea（18%）、Fatigue （12%）、Nauseous（11%）
Catumaxomab	NCT00836654	EPCAM × CD3	Puncture-free surviva in catumaxomab group (median 46 days) VS. in control group (median 11 days), $p < 0.0001$; Time to next paracentesis in catumaxomab group (77 days) VS. in control group (13 days), $p < 0.0001$.	TRAEs: 85%

TRAEs, treatment-related adverse events; ORR, objective response rate; DOR,duration of response; PFS, progression-free survival; OS, overall survival; tpCR, total pathological complete response; bpCR, breast pathological complete response; CBR, clinical benefit ratio; mPFS,median progression-free survival; DCR, disease control rate; CR,Complete Response; PR, partial response; SD, stable disease.

- Clinical trial registry records (NCT/ChiCTR numbers as listed).
